# The Spanish Version of the Unified Protocol for Transdiagnostic Treatment of Emotional Disorders in Adolescents (UP-A) Adapted as a School-Based Anxiety and Depression Prevention Program: Study Protocol for a Cluster Randomized Controlled Trial

**DOI:** 10.2196/resprot.7934

**Published:** 2017-08-21

**Authors:** Julia García-Escalera, Rosa M Valiente, Paloma Chorot, Jill Ehrenreich-May, Sarah M Kennedy, Bonifacio Sandín

**Affiliations:** ^1^ Faculty of Psychology Universidad Nacional de Educación a Distancia Madrid Spain; ^2^ Department of Psychology University of Miami Coral Gables, FL United States

**Keywords:** universal prevention, transdiagnostic, cluster, randomized controlled trial, unified protocol, adolescents, anxiety, depression, emotional disorders, school intervention

## Abstract

**Background:**

Anxiety and depression are common, impairing conditions that evidence high comorbidity rates in adolescence. The Unified Protocol for Transdiagnostic Treatment of Emotional Disorders in Adolescents (UP-A) is one of the few existing resources aimed at applying transdiagnostic treatment principles to target core dysfunctions associated with both anxiety and depression within a single protocol. To our knowledge, this is the first study examining the efficacy of the UP-A adapted as a universal preventive intervention program.

**Objective:**

The primary aim of this study is to examine whether the Spanish version of the UP-A is more effective than a waitlist (WL) control group in reducing and preventing symptoms of anxiety and depression when employed as a universal, classroom-based preventive intervention. The secondary aim is to investigate changes in a broad range of secondary outcome measures, including negative and positive affect, anxiety sensitivity, emotional avoidance, top problems ratings, school grades, depression and anxiety-related interference, self-esteem, life satisfaction, quality of life, conduct problems, hyperactivity/inattention symptoms, peer problems, prosocial behavior, school adjustment, and discipline problems. Other aims are to assess a range of possible predictors of intervention effects and to examine the feasibility and the acceptability of implementing UP-A in a prevention group format and in a school setting.

**Methods:**

A cluster, randomized, WL, controlled trial design with classroom as the unit of randomization was used in this study. Five classes including a total of 152 adolescents were randomized to the experimental or WL control groups. Participants in the experimental group received 9 55-minute sessions delivered by advanced doctoral and masters students in clinical psychology. The WL control group will receive the intervention once the 3-month follow-up assessment is completed.

**Results:**

We have recruited participants to the cluster randomized controlled trial (RCT) and have conducted the intervention with the experimental group. We expect the WL control group to complete the intervention in July 2017. Data analysis will take place during the second semester of 2017.

**Conclusions:**

We expect the experimental group to outperform the WL control group at post-intervention and 3-month follow-up. We also expect the WL control group to show improvements in primary and secondary outcome measures after receiving the intervention. Results will have implications for researchers, families, and education providers.

**Trial Registration:**

Clinicaltrials.gov NCT03123991; https://clinicaltrials.gov/ct2/show/NCT03123991 (Archived by WebCite at http://www.webcitation.org/6qp7GIzcR)

## Introduction

### Background

Depression and anxiety disorders are highly prevalent conditions in children and adolescents and are associated with significant impairment in everyday life [1,2]. For instance, a national study conducted in the United States including adolescents aged 13 to 18 years found that anxiety disorders were the most common condition (31.9%) and that mood disorders were also highly prevalent (14.3%) [2]. In Spain, after conduct disorders, depressive disorders are the most prevalent psychological disorders in children and adolescents (14.6%), followed closely by anxiety disorders (13.3%) [1].

The costs of supporting children and adolescents with psychiatric disorders can be far higher than for their peers without these problems, and these disorders usually lead to continued financial burden and functional impairment into adulthood (eg, increased use of public sector services, lower-quality peer and family relationships, and reduced participation in the labor market) [3]. As an example, a Dutch cost-of-illness study focusing on 118 anxious 8 to 18-year old youth concluded that “the societal costs of families with a clinically anxious child who seek treatment amount to more than 20 million Euros a year in the Dutch population, and were 21 times higher than in families of the general population” [4]. Beyond the significant emotional and financial costs of childhood emotional disorders, depression and anxiety disorders are associated with increased risk for a range of difficulties throughout adolescence and into adulthood. Specifically, adolescent anxiety and depression are associated with an increased risk of educational under-achievement, substance dependence, chronic anxiety and/or depression, as well as impairment in a range of important psychosocial domains including interpersonal functioning, quality of life, and physical well-being [5,6].

Despite this chronic and concerning level of impairment, only about 20% of youth with anxiety or depressive disorders receive mental health services [7]. Given the sequelae of negative outcomes associated with childhood emotional disorders, preventative interventions are all the more important for their potential to reduce and even eliminate the distress, long-term impairment, and significant societal burden these disorders cause. Moreover, delivering universal prevention programs for anxiety and depression in a school setting may be an ideal method for making preventative interventions more widely accessible to those with a high risk of developing emotional disorders.

Prevention programs may be universal, selected, or indicated [8]. A universal approach to anxiety and depression prevention in schools could be especially beneficial because such an approach “seeks to target a large number of children regardless of their risk status over a short period of time, helps to reduce difficulties in screening for inclusion, potentially reduces the incidence of anxiety disorders by intervening before the onset of these disorders, and serves to reduce stigmatization” [9]. Preventive interventions have often been developed separately for anxiety and for depression and have typically considered these disorder classes as independent constructs [10]. However, anxiety and depressive disorders are frequently comorbid in children and adolescents, with studies reporting that about 25% to 50% of depressed youth have comorbid anxiety disorders and about 10% to 15% of anxious youth have comorbid depression [11]. In addition, both clinical manifestations share vulnerability and maintenance factors such as neuroticism or high negative affect, maladaptive action tendencies (eg, avoidance or withdrawal), and high levels of distress or discomfort in response to emotional experiences [12,13].

These findings suggest that a transdiagnostic approach to the prevention of adolescent anxiety and depression may be useful. Transdiagnostic cognitive behavioral therapy (T-CBT) has been defined as a type of cognitive behavioral therapy (CBT) that can be applied to a number of different disorders that share commonalities in cognitive, behavioral, and/or emotional dysregulation [14], without tailoring the protocol to specific diagnoses [15]. Fairburn, Cooper, and Shafran [16] initially developed and evaluated a transdiagnostic approach to CBT for eating disorders. Research on the development and efficacy of transdiagnostic interventions was advanced by Barlow and colleagues, who provided a transdiagnostic theoretical model and developed a unified (transdiagnostic) treatment for emotional disorders among adults: The Unified Protocol for Transdiagnostic Treatment of Emotional Disorders (UP) [17]. Drawing from research with the UP in adult samples, Ehrenreich-May and colleagues developed the Unified Protocol for Transdiagnostic Treatment of Emotional Disorders in Adolescents (UP-A) and the Unified Protocol for Transdiagnostic Treatment of Emotional Disorders in Children (UP-C) [18]. Preliminary data suggest that both protocols are effective in reducing principal and overall emotional disorder severity [19-21] and, overall, T-CBT for children and adolescents has demonstrated promising results according to a recent meta-analysis [21].

Applying a transdiagnostic approach to prevention that focuses on core dysfunctions across emotional disorders may reap a larger benefit than intervening with risk factors specific to only one disorder or domain type (eg, anxiety versus depression, etc) [10]. However, transdiagnostic, theory-driven CBT protocols for preventing anxiety and depression are still scarce, currently with only the following 2 well-consolidated protocols: (1) EMOTION: “Coping Kids” Managing Anxiety and Depression [22]; and (2) Super Skills for Life [23]. Studies applying these T-CBT-based prevention protocols have shown promising results [9,24]. In addition, a transdiagnostic, school-based preventive intervention for adolescents with elevated symptoms of social anxiety and/or depression and elevated peer victimization was developed and initially evaluated [25]. However, the EMOTION and the Super Skills for Life protocols were developed for young children [22,23], and T-CBT preventive interventions for anxiety and depression in adolescents are scarce, to say the least. An initial version of the UP-C was developed as a universal preventive intervention in a summer camp setting [26], providing useful preliminary data on the feasibility and efficacy of adapting and delivering the UP-C and UP-A as preventive interventions, but larger studies are needed, particularly in adolescents for whom T-CBT preventive interventions are lacking.

### Study Aims

The current study proposes to extend transdiagnostic prevention research for adolescents by establishing initial pre- to post-prevention and follow-up outcomes associated with the use of the Spanish version of the UP-A, adapted as a school-based anxiety and depression preventive intervention.

The primary aim of this study is to examine whether the UP-A, a T-CBT intervention adapted as a school-based preventive intervention and delivered by doctoral and masters students in clinical psychology, is more effective than a waitlist (WL) control condition consisting of the usual academic curriculum in (1) reducing symptoms of anxiety and depression; and (2) preventing clinically elevated levels of anxiety and depression from developing. The second aim is to investigate post-intervention and follow-up changes in a broad range of secondary outcome measures, including negative and positive affect, anxiety sensitivity, emotional avoidance, depression and anxiety-related interference, top problems ratings, school grades, self-esteem, life satisfaction, quality of life, school adjustment, discipline problems, conduct problems, hyperactivity/inattention symptoms, peer problems, and prosocial behavior. The third aim is to assess whether any benefits of the intervention are predicted by age, gender, number of sessions attended, participant-rated behaviors indicating level of engagement and effort during sessions, understanding of basic program concepts, adherence to home practice, practice of specific strategies outside session, and interest in psychology. The fourth aim is to examine if the effects at post-intervention are maintained at the 3-month follow-up. The fifth aim of this study is to assess the feasibility (eg, obtaining consent, assessment completion, group attendance) and the acceptability of implementing UP-A in a prevention group format and in a school setting. Finally, the sixth aim is to calculate the intracluster correlation coefficient (ICC) and measures of variability (ie, standard deviation, interquartile range) for all primary and secondary outcomes in order to determine sample sizes of future full-scale, cluster randomized controlled trials (RCTs) applying the UP-A adapted as a preventive intervention.

We predict that the UP-A preventive intervention group will exhibit greater improvement on all primary and secondary outcome measures at post-intervention and follow-up compared to the WL control group. We also expect that WL participants will experience significant improvements on all outcome measures after receiving the intervention. Greater improvement in the primary and secondary outcome variables is expected in those with higher levels of home practice, more frequent practice of a greater number of strategies outside session, higher levels of engagement and effort during sessions, more sessions attended, greater knowledge of core program contents after the intervention, and greater interest in psychology. In relation to age and gender as predictors, earlier studies have shown contradictory results [27] and therefore no hypothesis was forwarded in this regard. We also hypothesize that results will support the feasibility of school-based implementation of UP-A in a prevention group format, as evidenced by a number of participants achieving treatment completer status (attending at least 7 out of the 9 sessions), as well as the acceptability of the intervention, as evidenced by participants’ self-rated satisfaction at post-intervention.

## Methods

The study design reported is in line with the Consolidated Standards of Reporting Trials (CONSORT) statement: extension to cluster randomized trials [28]. The study was granted ethical approval from the Research Ethics Committee of Universidad Nacional de Educación a Distancia, Madrid, Spain. All parents or guardians as well as adolescent participants provided written informed consent. The study is registered in Clinicaltrials.gov (NCT03123991).

### Study Design

**Figure 1:**
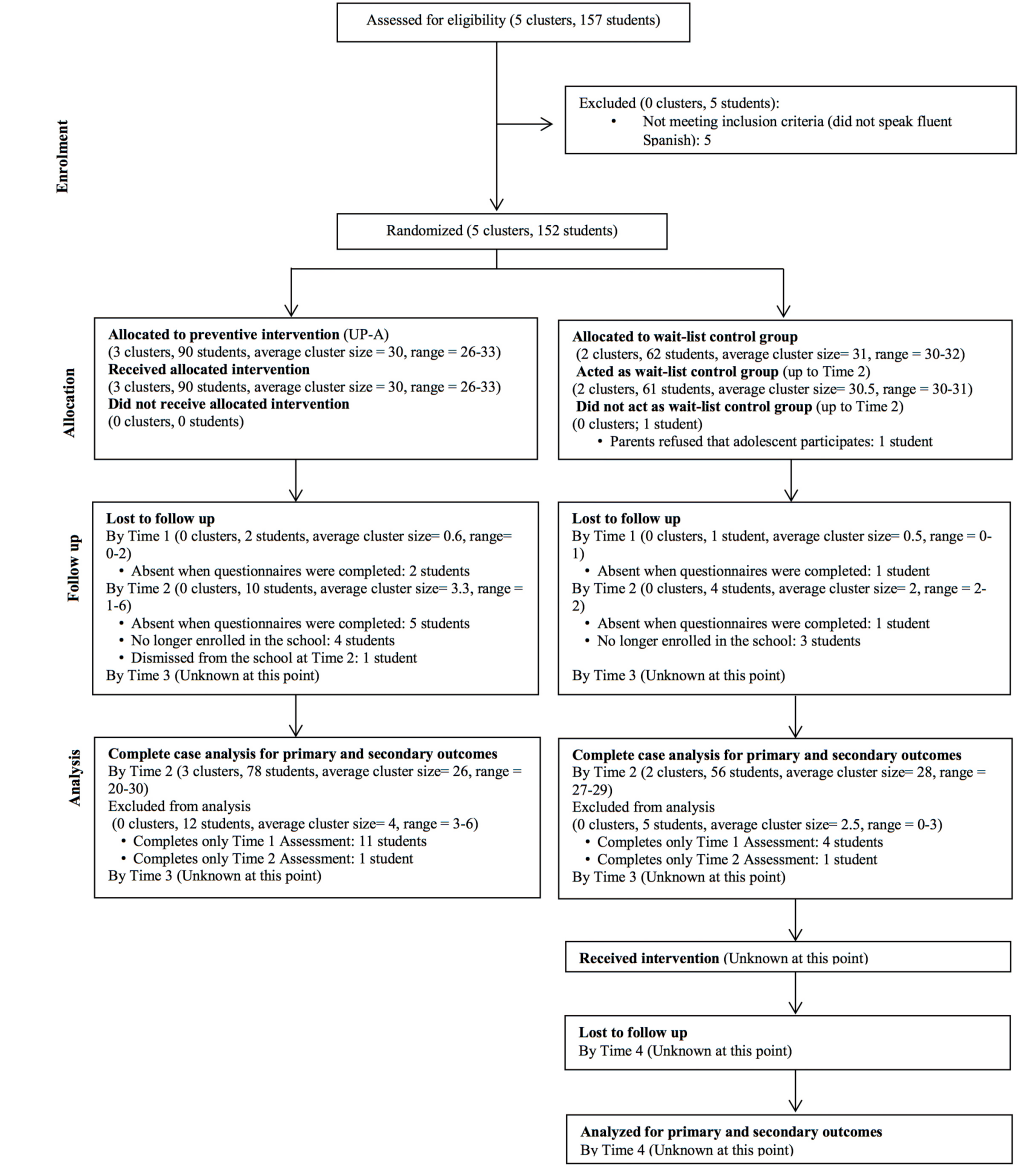
CONSORT flow diagram.

This study will be implemented as a 2-arm, cluster RCT [28], with an intervention condition (UP-A adapted as a preventive intervention program) and a 3-month WL control condition. Measurements will be taken on the following occasions: (1) Time 1 (1 week before the experimental group starts the intervention); (2) Time 2 (1 week after the experimental group finishes the intervention); (3) Time 3 (3 months after the experimental group finishes the intervention and 1 week before the WL control group starts the intervention); and (4) Time 4 (1 week after the WL control group finishes the intervention). On all occasions, except for Time 4, both groups will complete the outcome measures at about the same time. For Time 4, only the WL control group will complete the outcome measures. This represents a 3 (time) by 2 (group) repeated measures design. The flow diagram of the study is shown in Figure 1.

### Participants

An urban secondary school in the city of Madrid (Spain) that was previously known to the authors expressed interest in being involved in research and agreed to participate. Students enrolled in levels 3º ESO (usually aged 14 to 15 years old) and 4º ESO (usually aged 15 to 16 years old) were targeted, given that early to mid-adolescence is a key time for the emergence of common mental health disorders [29]. Participants came from 3 classes at level 3º ESO and 2 classes at level 4º ESO (each class having around 30 students each). No incentives are being given to the adolescents or the school for participating in this project.

Parents or guardians, as well as adolescents, were provided with information about the study and were required to provide signed consent before Time 1 assessments. Participants and families were informed that (1) responses to the questionnaires will be keep confidential; (2) they will be able to withdraw from the study at any time; (3) participants receiving the preventive intervention will be taught specific skills that will help them to better cope with situations that provoke anxiety/depression both now and in the future; and (4) participants assigned to the WL control condition will receive the preventive intervention after the WL period. Inclusion criteria for participants were providing written, informed consent (both the adolescent and at least 1 parent or legal guardian) and being able to understand, write, and read Spanish. Spanish proficiency was determined based on teacher report. Due to the universal prevention goal of this study, there were no other exclusion criteria. Classes of students were randomly assigned either to the preventive intervention group or to the WL control group (see Figure 1). Time 1, Time 2, Time 3, and Time 4 assessments, as well as intervention sessions, are being conducted at the school during school hours.

### Intervention

Participants will receive the Spanish version of the UP-A [18], modified for delivery as a 9-session, school-based universal preventive intervention. Core modules of the UP-A include (1) Building and Keeping Motivation; (2) Getting to Know Your Emotions and Behaviors; (3) Emotion-Focused Behavioral Experiments; (4) Awareness of Physical Sensations; (5) Being Flexible in Your Thinking; (6) Awareness of Emotional Experiences; (7) Situational Emotion Exposures; and (8) Keeping it Going-Maintaining Your Gains [18] (Figure 1).

The UP-A is a downward extension of the UP [17], modified for adolescents (ages 12 to 17) presenting with any primary emotional disorder, including anxiety, depressive, obsessive-compulsive, and stress-related disorders and problem areas. The UP-A distills common evidence-based techniques that cut across disorder-specific treatment manuals for youth emotional disorders (eg, psychoeducation, non-judgmental awareness, cognitive reappraisal, exposure, behavioral activation, etc) [30], drawing from CBTs, motivational enhancement, and third-wave behavioral therapies (eg, acceptance and mindfulness-based techniques). As with the UP, the UP-A emphasizes 5 core treatment principles: (1) increasing present-focused awareness of emotions; (2) enhancing cognitive flexibility; (3) identifying and preventing emotional avoidance and maladaptive emotion-driven behaviors; (4) increasing emotional awareness and acceptance of uncomfortable emotion-related physiological sensations; and (5) facilitating exposure to both interoceptive and situational triggers of emotional experiences [17,31].

The preventive intervention applied in this study consists of 9 weekly lessons, the length of which corresponds to a school's typical class period (55 minutes in the school in our study). It is delivered in a group format to entire classes of adolescents as part of the school curriculum. Specifically, the intervention sessions are carried out during school hours designated for “Tutorías,” which are 1-hour weekly sessions that, in the Spanish Education System, are meant to target issues occurring within the school context, such as providing professional development, providing academic support, assisting in solving problems between students or between students and teachers, etc. The WL control group will receive their normal class schedule without any planned socioemotional focus, followed by the intervention after the Time 3 assessment is completed. A detailed description of the content of each UP-A session is shown in Table 1.

The students in the preventive intervention group are encouraged to practice skills learned in sessions by completing structured home learning assignments outside of formal session time. Completed home learning assignments are discussed at the beginning of each session, with the exception of the first. All intervention sessions are delivered by author JGE, an advanced doctoral student in clinical psychology, and by an advanced master's student in clinical psychology.

Researchers attempt to contact students who miss one of the weekly sessions and provide them with the opportunity to make up the content in the following days. During this makeup session, students are given a content summary and any missed home learning assignments to facilitate preparation for the next session. If a student is unable to be contacted, he or she is able participate in the following session after a brief, individual content review meeting with one of the group leaders delivering the sessions.

### Implementation of the Program

Prior to implementing the UP-A program, JGE received training on the UP-A protocol by its developer, JEM at University of Miami (Coral Gables, US). Likewise, RMV, PC, and BS received specific training on the UP-A through a course on the UP-A and UP-C protocols at Universidad Nacional de Educación a Distancia (Madrid, Spain) given by JEM. The UP-A was translated into Spanish by JGE, and its translation and adaptation were supervised by BS, PC, and RMV. The translation process was also supervised by JEM. Adherence to the protocol for the current study is self-monitored by the group leaders, who complete a checklist at the end of each session indicating whether each skill within the session was presented.

### Primary Outcome Measures

Questionnaires will be completed at the Time 1, Time 2, Time 3, and Time 4 assessments (Table 2). Top Problems Assessment (TPA) is not included in this table because it was completed at time-points during the 1st, 5th, and 9th sessions (see below).

**Table 1:** The Unified Protocol for Transdiagnostic Treatment of Emotional Disorders in Adolescents (UP-A) preventive intervention session descriptions.

Session	UP-A^a^ module	Module title	Main content
1	Module 1	Building and Keeping Motivation	Introduce confidentiality and group rules; obtain 3 top problems, severity ratings, and a SMART^b^ goal for each problem; complete emotion identification skills activity if sufficient time remains.
2	Module 2	Getting to Know Your Emotions and Behaviors	Psychoeducation about emotions and their function; introduce emotional behaviors, the 3 parts of an emotional experience, and the “Before, During, and After” form for tracking emotional experiences outside of sessions.
3	Module 3	Emotion-Focused Behavioral Experiments	Psychoeducation about cycle of avoidance, opposite action, and behavioral experiments; reflect on current use of free time and come up with a list of enjoyed activities; introduce weekly activity tracker for on-going behavioral activation.
4	Module 4	Awareness of Physical Sensations	Psychoeducation about body sensations, their relationship to intense emotions and their harmlessness; introduce the concept of “fight or flight response” and review cycle of avoidance; conduct sensational exposures with the group.
5	Module 5	Being Flexible in Your Thinking	Introduce the concept of “thinking traps” (ie, cognitive distortions) and teach common thinking traps; introduce the concept of automatic and alternative thoughts as well as detective thinking skills; Re-rate top problems obtained in session 1.
6	Module 5	Being Flexible in Your Thinking	Review thinking traps and detective thinking skills; introduce and ensure understanding of problem solving skills; conduct examples using problem solving skills with group members; review skills learnt so far in the program.
7	Module 6	Awareness of Emotional Experiences	Introduce the rationale for present-moment awareness and practice this skill in session using non-emotional stimuli (eg, focus on breathing); introduce rationale for non-judgmental awareness; do an individual mini-test assessing skills taught in the program so far.
8	Module 7	Situational Emotion Exposures	Review cycle of avoidance, reinforcement, and maintenance of learned behavior; provide psychoeducation about emotion exposures; create emotional behaviors forms to identify relevant exposures; if time permits, conduct a group exposure activity; assign exposure homework.
9	Module 8	Keeping it Going—Maintaining Your Gains	Review exposure homework and plan future exposures if necessary; re-rate top problems and revisit SMART goals; review skills that have been most useful for each group member and make an individualized post-program plan to practice skills.

^a^UP-A: Unified Protocol for Transdiagnostic Treatment of Emotional Disorders in Adolescents.

^b^SMART: specific, measurable, attainable, relevant, and time-bound.

**Table 2:** Outcome measures used in the study.

Questionnaire	Assessment								Items, n
	Time 1		Time 2		Time 3		Time 4		
	E^a^	WL^b^	E	WL	E	WL	E	WL	
Demographics^c^	Yes	Yes	Yes	Yes	Yes	Yes	No	Yes	9/20
RCADS-30^d^	Yes	Yes	Yes	Yes	Yes	Yes	No	Yes	30
CDN^e^	Yes	Yes	Yes	Yes	Yes	Yes	No	Yes	14
EAN^f^	Yes	Yes	Yes	Yes	Yes	Yes	No	Yes	10
SDQ^g^	Yes	Yes	Yes	Yes	Yes	Yes	No	Yes	25
EIDAN^h^	Yes	Yes	Yes	Yes	Yes	Yes	No	Yes	11
PANASN^i^	Yes	Yes	Yes	Yes	Yes	Yes	No	Yes	20
CASI^j^	Yes	Yes	Yes	Yes	Yes	Yes	No	Yes	18
EASI-A^k^	Yes	Yes	Yes	Yes	Yes	Yes	No	Yes	17
SWLS-C^l^	Yes	Yes	Yes	Yes	Yes	Yes	No	Yes	5
KIDSCREEN-10^m^	Yes	Yes	Yes	Yes	Yes	Yes	No	Yes	10
SES^n^	Yes	Yes	Yes	Yes	Yes	Yes	No	Yes	10
EBAE-10^o^	Yes	Yes	Yes	Yes	Yes	Yes	No	Yes	10
IG^p^	Yes	Yes	Yes	Yes	Yes	Yes	No	Yes	10
Satisfaction Q^q^	No	No	Yes	No	No	No	No	Yes	10
Curriculum Q^r^	No	No	Yes	No	Yes	No	No	Yes	3
Strategies Q^s^	No	No	Yes	No	Yes	No	No	Yes	8
Discipline Q^t^	No	No	Yes	No	No	No	No	Yes	6
Other questions^u^	No	No	Yes	No	No	No	No	Yes	4
Psychology Q^v^	No	No	Yes	Yes	Yes	Yes	No	Yes	3
PID-5-Brief^w^	No	No	Yes	Yes	No	No	No	No	25

^a^E: experimental group.

^b^WL: waitlist control group.

^c^The demographics questionnaire has 20 items at Time 1 and 9 items at Time 2, Time 3, and Time 4.

^d^RCADS-30: Revised Child Anxiety and Depression Scale-30.

^e^CDN: Depression Questionnaire for Children (Cuestionario de Depresión para Niños).

^f^EAN: Anxiety Scale for Children (Escala de Ansiedad para Niños).

^g^SDQ: Strengths and Difficulties Questionnaire.

^h^EIDAN: Depression and Anxiety Interference Scale for Children (Escala de Interferencia de la Depresión y Ansiedad para Niños).

^i^PANASN: Positive and Negative Affect Schedule for Children and Adolescents (Escalas PANAS de Afecto Positivo y Negativo para Niños y Adolescentes).

^j^CASI: Childhood Anxiety Sensitivity Index.

^k^EASI-A: Emotional Avoidance Strategy Inventory for Adolescents.

^l^SWLS-C: Satisfaction with Life Scale for Children.

^m^KIDSCREEN-10: Kidscreen-10 Quality of Life Scale.

^n^SES: Self-Esteem Scale.

^o^EBAE-10: School Adjustment Brief Scale (Escala Breve de Ajuste Escolar).

^p^IG: General Indiscipline Scale (Escala de Indisciplina General).

^q^Satisfaction Q: satisfaction with the program questionnaire.

^r^Curriculum Q: curriculum knowledge questionnaire.

^s^Strategies Q: strategies practiced outside of session questionnaire.

^t^Discipline Q: discipline problems during sessions questionnaire.

^u^Other questions: other end of program questions.

^v^Psychology Q: assistance to therapy and interest for psychology questions.

^w^PID-5-Brief: Personality Inventory for Diagnostic and Statistical Manual of Mental Disorders, 5th edition (DSM-5)-Brief Form.

#### Revised Child Anxiety and Depression Scale-30

The Revised Child Anxiety and Depression Scale-30 (RCADS-30) [32] is a widely-used questionnaire measuring self-reported anxiety and depressive symptoms in children and adolescents. The scale is comprised of the following subscales derived from the Diagnostic and Statistical Manual of Mental Disorders, 4th edition (DSM-IV) criteria: (1) social phobia, (2) generalized anxiety disorder, (3) obsessive-compulsive disorder, (4) panic disorder, (5) separation anxiety disorder, and (6) major depressive disorder. The 6 subscales are summed to create a Total Anxiety and Depression score. Each item is scored from 0 (“Never”) to 3 (“Always”), with higher scores representing more severe symptoms. The RCADS-30 has demonstrated good psychometric properties with normative and clinical populations [33,34]. In the current sample, the 6 individual subscales have demonstrated adequate to good internal consistency both at Time 1 (alpha .70 to .85) and at Time 2 (alpha .72 to .86). The Total Anxiety and Depression scale has demonstrated excellent internal consistency at Time 1 (alpha = .92) and at Time 2 (alpha = .93) assessments.

#### Depression Questionnaire for Children

Depression Questionnaire for Children (Cuestionario de Depresión para Niños; CDN) [35] is a 16-item, self-report questionnaire designed to assess symptoms of DSM-IV major depressive disorder and dysthymic disorder in children and adolescents. In this study, the 2 items targeting suicidal ideation were not included as per the request of school personnel. Participants rate each item on a 3-point scale from 0 (“Never”), 1 (“Sometimes”), to 2 (“Very often”) to indicate the frequency with which they experience depression symptoms. The sum of all items provides an overall score, with higher scores indicating greater depression symptoms. The CDN has demonstrated adequate psychometric properties [32]. At Time 1 and Time 2 assessments, the CDN overall scale has demonstrated good internal consistency in this sample with alpha values of .87 and .89, respectively.

#### Anxiety Scale for Children

Anxiety Scale for Children (Escala de Ansiedad para Niños; EAN) [35] is a 10-item questionnaire that assesses anxiety symptoms during the past few weeks in children and adolescents. Participants are instructed to indicate how frequently they have experienced general anxiety symptoms on a 4-point, Likert-type scale, ranging from 0 (“Never or almost never”) to 3 (“A lot of the times or almost always”). The sum of all items provides an overall score, with higher scores indicating more elevated anxiety symptoms. The EAN has shown good psychometric properties [36]. In the current sample, the EAN scale has demonstrated excellent internal consistency at Time 1 (alpha = .91) and Time 2 (alpha = .94) assessments.

### Secondary Outcome Measures

The following questionnaires will be completed at Time 1, Time 2, Time 3, and Time 4 assessments (see Table 2).

#### Top Problems Assessment

The adolescent version of TPA provides a means of collecting information about changes in the severity of problems identified by the adolescent to be of greatest concern. Group leaders first provide instructions and examples about the types of problems that can be targeted within the program and adolescents are then asked to generate 3 top problems of their own. Adolescents also generate a corresponding specific, measurable, attainable, relevant, and time-bound (SMART) goal for each problem. During the 1st, 5th, and 9th sessions, participants are asked to rate the severity of each problem on a 0 to 8 scale (with higher ratings indicating greater problem severity). Top problems are a central tool for progress monitoring in the UP-A and UP-C protocols. They were adapted by Ehrenreich-May from original work by Weisz and colleagues [37]. Top problems have been shown to demonstrate good psychometric properties [37].

#### Strengths and Difficulties Questionnaire

The Spanish version of the Strengths and Difficulties Questionnaire (SDQ) by García and colleagues was used [38,39]. The scale provides scores for 5 subscales including emotional symptoms, conduct problems, symptoms of hyperactivity/ inattention, peer problems, and prosocial behavior. Each subscale contains 5 items scored on a 3-point, Likert-type scale, ranging from 0 (“Not true”) to 2 (“Certainly true”). A Total Difficulties Score is obtained by summing the 5 items on all scales, with the exception of the prosocial behavior scale. The SDQ is widely used and both the original and Spanish versions have good psychometric properties [40,41]. The Total Difficulties Scale has demonstrated adequate to good internal consistency in this sample at Time 1 (alpha = .73) and Time 2 (alpha = .81) assessments.

#### Depression and Anxiety Interference Scale for Children

Students complete a Depression and Anxiety Interference Scale for Children (Escala de Interferencia de la Depresión y Ansiedad para Niños; EIDAN) [42] developed for this investigation. This 11-item questionnaire assesses the degree to which feeling worried, nervous, or sad interferes with various domains of the adolescent’s life (school, peer/family/general functioning), employing a 4-point, Likert-type scale ranging from 0 (“Nothing or almost nothing”) to 3 (“A lot”). Higher scores indicate greater levels of interference. In the current sample, the EIDAN scale has demonstrated good internal consistency at Time 1 (alpha = .80) and Time 2 (alpha = .87) assessments.

#### Positive and Negative Affect Schedule for Children and Adolescents

The Positive and Negative Affect Schedule for Children and Adolescents (Escalas PANAS de Afecto Positivo y Negativo para Niños y Adolescentes; PANASN) [43] questionnaire is an age-downward version of the Positive and Negative Affect Schedule [44] for individuals aged 7 to 17 years. The scale provides scores for 2 subscales of 10 items each measuring positive and negative affect. Participants are asked to rate items according to how they usually feel from 1 (“Never or almost never”), 2 (“Sometimes”), to 3 (“A lot of the time”). The PANASN has demonstrated good psychometric properties [43,45]. In the current sample, the Positive Affect Scale demonstrated adequate and good psychometric properties at Time 1 (alpha = .77) and Time 2 (alpha =.81) assessments. Similarly, the Negative Affect Scale demonstrated adequate internal consistency at Time 1 assessment (alpha = .79) and good internal consistency at Time 2 assessment (alpha = .81).

#### Childhood Anxiety Sensitivity Index

The Spanish version of the Childhood Anxiety Sensitivity Index (CASI) [46,47] was used for this study. The CASI is an 18-item self-report questionnaire measuring anxiety sensitivity in children or distress reactions to symptoms of anxiety (eg, “It scares me when my heart beats fast”). Participants rate the frequency with which they experience each item using a 3-point, Likert-type scale from 1 (“Never”), 2 (“Sometimes”), to 3 (“A lot of the time”). The Spanish adaptation of the CASI used in the present study has demonstrated good psychometric properties and positive correlations with constructs related to anxiety sensitivity [48]. In the current sample, the CASI has demonstrated excellent internal consistency at Time 1 (alpha = .90) and Time 2 (alpha = .90) assessments.

#### Emotional Avoidance Strategy Inventory for Adolescents

Emotional Avoidance Strategy Inventory for Adolescents (EASI-A) [49] is a 17-item self-report questionnaire in which respondents are instructed to indicate the degree to which each statement is true using a 5-point, Likert-type scale ranging from 0 (“Not at all true of me”) to 4 (“Extremely true of me”). The EASI-A was translated and adapted to Spanish for this study [50]. Our Spanish adaptation also uses a 5-point Likert-type scale, but with options indicating frequency rather than degree from 0 (“Never or almost never”), 1 (“Seldom”), 2 (“Sometimes”), 3 (“A lot of times”), to 4 (“Always or almost always”). The EASI-A has demonstrated good psychometric properties and positive correlations with anxiety and depression symptoms [49]. The EASI scale has demonstrated good internal consistency at Time 1 and Time 2 assessments with alpha values of .87. and .86, respectively, in the current sample.

#### Satisfaction with Life Scale for Children

The Satisfaction with Life Scale for Children (SWLS-C) [51] measure is an age-downward version of the measure of life satisfaction developed by Diener and colleagues [52]. It is a 5-item, self-report instrument in which respondents are asked to indicate the degree to which each statement is true of their life using a 4-point, Likert-type scale ranging from 1 (“Not at all”) to 4 (“A lot or completely”). In the current sample, the SWLS scale has demonstrated adequate good consistency at Time 1 (alpha = .81) and Time 2 (alpha = .89) assessments.

#### Kidscreen-10

The official Spanish version developed by the KIDSCREEN Group was used [53]. KIDSCREEN-10 is a widely-used brief questionnaire assessing generic health-related quality of life in children and adolescents that was adapted from the longer KIDSCREEN-27 [53]. The 10 items on the KIDSCREEN-10 index assess affective symptoms of depressed mood, difficulty concentrating, decreased energy, impaired school functioning, and impaired relations with peers and family. It uses a 5-point, Likert-type scale ranging from 1 (“Not at all”) to 5 (“Extremely”). KIDSCREEN-10 has been shown to possess good psychometric properties [54]. In the current sample, the KIDSCREEN-10 scale has demonstrated good internal consistency at Time 1 (alpha =.82) and Time 2 (alpha = .85) assessments.

#### Self-Esteem Scale

A Spanish adaptation of the Self-Esteem Scale (SES) [55] was used in this study [56]. SES is a 10-item self-report instrument that measures global self-esteem. Respondents are asked to indicate how much they agree with each statement on a 4-point, Likert-type scale from 1 (“Strongly disagree”) to 4 (“Strongly agree”). The scale has been shown to possess good psychometric properties in adolescent samples [57]. In the current sample, the SES scale has demonstrated good internal consistency at Time 1 (alpha = .83) and excellent internal consistency at Time 2 (alpha = .90) assessments.

#### School Adjustment Brief Scale

School Adjustment Brief Scale (Escala Breve de Ajuste escolar; EBAE-10) [58] is a 10-item questionnaire used to measure adaptive functioning in the area of school performance. Respondents are instructed to respond to questions about their grades, their relationships with teachers and peers, and their expectations regarding their educational future. They are asked to indicate how much they agree with each statement on a 6-point, Likert-type scale ranging from 1 (“Completely agree”) to 6 (“Completely disagree”). This scale has demonstrated adequate psychometric properties [59]. In the current sample, the EBAE scale has demonstrated acceptable internal consistency at Time 1 (alpha=.76) and Time 2 (alpha=.72) assessments.

#### General Indiscipline Scale

The General Indiscipline Scale (Escala de Indisciplina General; IG), adapted from Martín and colleagues’ questionnaire [60], is an 11-item questionnaire that assesses problematic behaviors of students in the classroom. Respondents are asked to indicate the frequency of each behavior stated in the items on a 4-circle bull's-eye, with each circle closer to the bulls-eye's center representing more frequent demonstration of the behavior. For this study, we adapted the scale such that adolescents were asked to rate the frequency with which they demonstrated each behavior on a 4-point, Likert-type scale with options being 0 (“Never or almost never”), 1 (“Only sometimes”), 2 (“Quite a few times”), or 3 (“A lot of the time”). The IG has demonstrated adequate properties [61]. In the current sample, this scale has demonstrated adequate internal consistency at Time 1 (alpha = .75) and good internal consistency at Time 2 (alpha= .80) assessments.

#### Socio-Demographic Information Questionnaire

All participants are asked to provide demographic information including their age, gender, school grades, socio-economic status, and place of birth.

### Other Secondary Outcome Measures

#### The Personality Inventory for DSM-5-Brief Form

The Spanish version of the Personality Inventory for Diagnostic and Statistical Manual of Mental Disorders, 5th edition-Brief Form (PID-5-BF) [62,63] was used. It assesses 5 personality trait domains (negative affect, detachment, antagonism, disinhibition, and psychoticism), with each trait domain consisting of 5 items to be rated on a 4-point scale from 0 (“Very false or often false”), 1 (“Sometimes or somewhat false”), 2 (“Sometimes or somewhat true”), to 3 (“Very true or often true”). All items are summed to derive an overall score, with higher scores indicating greater overall personality dysfunction. Similarly, higher scores in each trait domain indicate greater dysfunction in that personality trait domain. In the current sample, the PID-5-BF overall scale has demonstrated excellent internal consistency (alpha = .90).

#### Therapy and Psychology Questions

At Time 2, Time 3, and Time 4 assessments, students are also asked whether they have attended therapy in the last 3 months (and if yes, for how many sessions), as well as a question regarding their interest in psychology, rated on a 4-point, Likert-type scale from 1 (“None”) to 4 (“A lot”).

### Measures Completed at Post-Intervention Only

The following questionnaires are to be completed by students in the preventive intervention group at Time 2 and by students in the WL control group at Time 4 (Table 2).

#### Satisfaction with the Program Questionnaire

We used 6 of the 7 questions from Rapee and colleagues’ Satisfaction Questionnaire [64]. The 6 questions assess enjoyment of the program, amount learnt in the program, the effectiveness of the program in improving general life coping skills, likelihood of recommending the program to others, and the ability to cope with emotions before and after the program. We added one other question “Did this program help you to learn more about emotions and how they work?” All items are assessed on a 10-point scale from 1 (“Least or none”) to 10 (“A lot”), with the exception of questions related to recommending the program to others and whether this program increased knowledge about emotions and how they work. These latter 2 questions are assessed using a dichotomous, 2-point scale with 1 being “Yes” and 2 being “No.” Rapee and colleagues’ Satisfaction Questionnaire has showed adequate psychometric properties [24,64].

#### Discipline Problems During Sessions Questionnaire

Students are asked 6 questions about how often they demonstrated certain behaviors during the sessions using a 4-point, Likert-type scale from 1 (“In no or almost no sessions of the program“), 2 (“Only in some sessions of the program”), 3 (“In quite a lot of the sessions of the program”), to 4 (“In all or almost all sessions of the program”). Specifically, the items were (1) “I have talked to my classmates when I should not have”; (2) “I have paid attention to what the girls delivering the program were saying”; (3) “I have done things from other subjects during program sessions”; (4) “I have been reprimanded for my behavior”; (5) “I have taken the program seriously”; and (6) “I have tried to do my best when doing the in-class activities of the program.” In the current sample, this questionnaire has demonstrated only fair internal consistency at Time 2 assessment (alpha = .68).

#### Other End of Program Questions

Adolescents are also asked the following other questions related to the program: (1) “What did you like best about the program?”; (2) “What did you like worst?”; (3) “Being honest with yourself, are you going to make efforts in the future to apply the strategies that you learned in this program in your daily life?”; (4) “When you missed a session, did you read the summary of the session that was given to you?”; and (5) “When you missed a session, did you do the homework that was given to you?” Questions 1 and 2 are open-choice questions and question 3 is to be answered using a dichotomous scale with 1 being “Probably yes” and 2 being “Probably not.” Questions 4 and 5 are to be rated on a 3-point scale from 1 (”Most of the time yes”), 2 (“Most of the times no”), to 3 (“I did not miss any sessions”).

### Measures Completed at Post-Intervention and Follow-Up

The following questionnaires are to be completed by students in the preventive intervention group at Time 2 and Time 3 and by students in the WL control group at Time 4 (Table 2).

#### Curriculum Knowledge Questionnaire

A questionnaire was created on the basis of the program curriculum to assess participants’ knowledge of core information presented in the program. Specifically, there are 2 open-choice questions (“What are the three parts of an emotion?” and “What can you do when you are feeling sad or down to feel better?”) and one multiple choice question (“What is a thinking trap?”) with 3 answer choices (“It is a kind of unpleasant emotion”/“It is what happens when someone tries to trick us into thinking what they want”/“It is a thought that makes us feel unpleasant emotions”).

#### Strategies Practiced Outside of Session Questionnaire

We adapted the questionnaire for our study based on the format used in a previous study by Johnson and colleagues [65]. Students are asked “During the 9-week course, how often did you practice each of the following techniques outside of the lessons?”, are supplied with a list of techniques learned during the course, and are asked to rate how much they practiced each technique. Adolescents rate each item on a 5-point, Likert-type scale from 1 (“Never”), 2 (“Once or twice in total”), 3 (“Greater than twice in total but less than once a week”), 4 (“Once or twice each week”), to 5 (“Three times or more each week”), with higher scores indicating more frequent practice of strategies learned in the program. Specifically, participants are asked about their use of the following strategies: (1) “Identify the three parts of the emotion you are feeling (what you think, what you feel in your body and what you do)”; (2) “Plan for how long are you going to do school work and what pleasant activities are you going to do”; (3) “When you are sad or worried, do something that you like or value even if you do not feel like it”; (4) “Realize that you are falling into a thinking trap (eg, thinking the worst, ignoring the positive, etc) and try to change the thought to an alternative one that makes you feel better”; (5) “When you have a problem, think about all the possible solutions, then think about the good and bad things about each solution and, lastly, choose one of the solutions to try”; (6) “Try to focus in the present moment”; (7) “Meditate, that is, sit and try to focus in your breathing for a few minutes”; and (8) “Expose yourself little by little to those things that scare you or make you nervous because you know it is the only way to overcome your fears.” At Time 3, the question is re-worded to: “Since the end of the program at school, how often have you used the following strategies?” In the current sample, this questionnaire has demonstrated adequate internal consistency at Time 2 assessment (alpha = .82).

### Sample Size

One significant impact of the adoption of a cluster design is the comparatively large sample size requirement since, in contrast to individually randomized trials where inter-individual variation is the only source of variability, cluster studies involve both variation among individuals and variation among clusters. As a result, cluster studies must recruit a larger number of individuals in order to achieve power equivalent to that of an individually randomized trial [28]. The magnitude of this within-cluster dependence, which ultimately influences the eventual trial size, is quantified by the intracluster correlation coefficient (ICC) [66]. The ICC accounts for the extent to which responses of adolescents attending the same class (that is, sharing the same classroom, classmates, teacher, etc) are more likely to be similar compared with adolescents from a different class.

Power analyses were conducted using G*Power Version 3 software [67]. Sample size required to detect a Cohen *d* effect of .30 was estimated, based upon effect sizes reported in meta-analyses of school-based anxiety and depression preventive interventions conducted all over the world [68,69], including in Spain [70]. Calculations showed that with a power level of .80 and a significance level of alpha = .05, a total sample of N_Non-cluster_ of 74 was required to detect a significant effect. However, potential loss of power due to data clustering had to be considered in the sample size calculation. The impact of the ICC on the planned trial size depends on the so-called design effect, which can be calculated as 1+(*m*-1) ICC, with *m* referring to the number of participants recruited per cluster [66]. For this study, the ICC is unknown although, on the basis of prior school-based prevention research, the expected ICC for anxiety and depression-related outcomes is approximately 0.02 [27]. The anticipated average class (cluster) size for this study is 27 students meeting inclusion criteria, resulting in a design effect of 1.52. The design effect is then multiplied by the previously calculated N_Non-cluster_ [66], resulting in an estimated sample size for this trial of 112, considering the design effect. In addition, we estimated a dropout rate of 10% based on previous studies [65,71], resulting in an estimated total sample size of 123 (at least 62 students in each group). The total number of clusters required can be calculated dividing the estimated total sample size by the estimated number of participants recruited per cluster (123/27), resulting in 4.56 clusters [66]. Therefore, to achieve a sample of this size, a total of 157 students and 5 clusters were recruited.

As the flow diagram shows (Figure 1), the final number of participants matches these a priori computations closely: 90 students (10 lost at Time 2) were allocated to the preventive intervention group and 62 students (4 lost at Time 2) were assigned to the WL control group. In addition, this study will allow for calculation of the ICC and effect sizes for all primary and secondary outcomes, which could be very important in assisting future researchers in establishing the feasibility and ideal sample size for future full-scale cluster RCTs applying the UP-A as a preventive intervention.

### Randomization

#### Sequence Generation and Allocation Concealment

Each participating class (cluster) was randomly allocated 1:1 to the preventive intervention or WL control condition. We used a balanced design, resulting in about the same number of classes in each of the preventive intervention and WL control groups (Figure 1). No matching, blocking, or stratification took place. Cluster randomization was undertaken for the ecological validity of providing the intervention at the class level. The randomization was conducted by a researcher not involved in the current project by using a computer random number generator. Random assignment occurred before Time 1 measurements took place because the Research Ethics Committee that provided ethical approval for this study requested that the Informed Consent forms signed by parents/guardians and participants state whether the student was going to be in the experimental or the WL control group.

#### Implementation and Blinding

The adolescents complete all questionnaires using Qualtrics Survey software in a designated classroom, and a research assistant is available to provide assistance if necessary and to ensure independent responding. The research assistant is blind to the allocated treatment group at time of completing questionnaires to reduce risk of bias. Blinding of participants at the cluster or individual level is not possible for the ethical reasons explained above. Regardless, blinding participants at the cluster or the individual level after baseline would have been impossible due to the nature of the experimental intervention, which requires active participation from the preventive intervention group compared to no involvement or participation from the WL control group in the first phase of the study. An attention-control intervention would have been ideal but was beyond the scope of this study.

## Results

Analysis and presentation of data will be in accordance with CONSORT guidelines and, in particular, the extension to cluster randomized trials [28]. Statistical significance will be considered as a *P* value of less than .05, and statistical analysis will be mainly carried out using IBM Statistical Package for the Social Sciences, Version 24.0 (IBM SPSS). Data will be analyzed taking the clustering of students within classes into account.

To check for possible differences at pre-intervention between intervention and WL control groups, a chi-square test for nonparametric variables and a 2-tailed *t* test for continuous variables will be performed at the student-level and class-level on sociodemographic variables as well as on primary and secondary outcome measures. Complete case analyses (excluding participants with missing data), intent-to-treat analyses (including all randomized individuals), and completer status analyses (ie, participants that achieved completer status, that is, participants that attended to 7 or more sessions), will be conducted according to the initial allocation of classes to either preventive intervention or WL control groups. Results will be reported at cluster and individual levels, including information about the estimated effect size and its precision, as well as ICCs for each primary and secondary outcome.

In relation to missing data, we will follow the guidelines for analyzing and reporting cluster RCTs with missing data established by Fiero and et al [72] and Díaz and et al [73]. Specifically, we will (1) attempt to follow up on all randomized clusters and individuals in order to limit the extent of missing data; (2) report the number of clusters and individuals lost to follow-up, as well as numbers of missing values for each variable of interest, which will be important when deciding what analysis to use; (3) collect and report information about reasons for losses to follow-up and other missing values, which may help when deciding the plausibility of the missing-at-random assumption; (4) justify the choice of principal analysis, the missing data mechanism assumed, and the plausibility of these assumptions; (5) perform and report sensitivity analyses to explore the robustness of the trial results to departures from the missing data assumption made in the primary analysis; and (6) follow the CONSORT extension for cluster trials [28] to ensure comprehensive reporting and transparency of methods used. To investigate the potential effects of missing data, the baseline characteristics of the adolescents will be compared for those with and without missing data.

The effectiveness of the intervention will be assessed using complex samples procedures in SPSS as well as multi-level (or hierarchical) modeling that includes both fixed (intervention effects) and random (students in classrooms) effects. Experimental and WL control groups will be compared at 3 points in time: Time 1 (pre-treatment), Time 2 (post-treatment for intervention group), and Time 3 (3-month follow-up for intervention group). In addition, within-participant analyses will be conducted only with the WL control group at Time 4. ICCs will be calculated for all primary and secondary outcomes to compare the variation due to school class and the total variance.

Secondary analyses will include (1) repeating the primary analysis adjusting for any variables exhibiting significant imbalance at baseline to assess whether this influences the findings; (2) examining intervention changes for adolescents who scored above the clinical cut-off for anxiety and/or depression at Time 1; (3) investigating potential predictors of intervention effects including all participants (both intervention and WL control groups) after the WL receives UP-A; (4) comparing observed and expected attrition rates, as well as observed and expected ICCs; and (5) analyzing answers to checklists completed by group leaders.

At the time of submitting this manuscript, all participants have been recruited and we have conducted Time 1 and Time 2 assessments with the experimental and WL control groups. The experimental group has also completed the preventive intervention. We expect the WL control group to complete the preventive intervention and Time 4 assessments in July 2017.

## Discussion

### Principal Findings

This paper describes the study protocol of a cluster RCT testing the first adaptation of the UP-A as a school-based anxiety and depression preventive intervention. The primary outcomes are change in anxiety and depression symptoms. Secondary outcomes include change in participant-identified top problems ratings, conduct problems, hyperactivity/inattention symptoms, peer problems, prosocial behavior, school grades, depression and anxiety-related interference, positive and negative affect, anxiety sensitivity, emotional avoidance, life satisfaction, quality of life, self-esteem, school adjustment, and discipline problems. This study will also improve our understanding of factors that might influence the effectiveness of UP-A through the preliminary examination of a number of potential predictors of treatment outcomes, including age, gender, number of sessions attended, engagement and effort during sessions, understanding of basic program concepts, adherence to home practice, and practice of specific strategies outside of sessions.

This study focuses on a school-based, universal prevention program for anxiety and depression, which has the advantage of being easily integrated into a school curriculum and thereby limiting the extent to which participants are segregated from peers and at risk for possible stigmatization due to their participation [74]. It is surprising that, despite the high co-occurrence between anxiety and depression and their shared risk and vulnerability factors, preventive interventions have typically focused on either depression or on anxiety alone [10]. For instance, of the 30 total studies included in a meta-analysis conducted by Ahlen and colleagues, 13 studies focused primarily on depression prevention, 10 studies focused primarily on anxiety prevention, and only 7 studies focused on prevention of both disorders [27]. A transdiagnostic approach to preventing anxiety and depression may enhance the efficacy, generalizability, and cost-effectiveness of prevention programs [10], as well as prevent the development of related emotional disorders.

The location of this research in Spain is particularly significant, as there is a lack of anxiety and depression preventive interventions for youth in the country [70]. A very recent systematic review and meta-analysis on school-based depression and anxiety prevention programs for young people [75] only found 2 studies that took place in Spain, one of them applying a selective preventive intervention and the other applying a universal one. In addition, the current organization of public healthcare in Spain is such that citizens lack direct access to psychological care services due to lengthy waiting lists and a limited number of psychologists [76], although there are some recent initiatives targeting adult populations that aim to implement evidence-based psychological treatments and increase the number of clinical psychologists in primary care [76,77]. Given the high need for mental health services in Spain relative to the number of existing providers, and given the high individual, familial, and societal burden of untreated or undertreated mental health concerns, the development of new and effective preventive intervention programs in the country is crucial.

### Strengths

The greatest strength of this study is that it is the first study to evaluate the effectiveness of the UP-A adapted as a school-based, universal anxiety and depression preventive intervention. In addition, it includes random assignment of clusters (classes) to intervention or WL conditions, implements an evidence-based protocol (the UP-A), uses highly reliable assessment measures, and includes a brief but reasonable window for follow-up after prevention programming (3 months). Another strength of this study is that we are not only examining the effectiveness of the UP-A program in reducing anxiety and depressive symptoms, but we are also examining its impact on a range of other variables. These variables include positive outcomes (quality of life, quality of peer relationships, prosocial behavior, self-esteem, satisfaction with life), which are especially important to measure when working at the universal prevention level, as the majority of participants tend to exhibit non-clinical level concerns [71]. We have also included a measure of personality traits, as the presence of certain personality traits has been identified as a potentially useful means of identifying individuals at risk for developing emotional disorders [78]. Furthermore, this study will not only evaluate the effectiveness of the UP-A program for the total sample of participating students, but it will also conduct preliminary analyses to investigate whether the program is more beneficial for certain groups of adolescents than others (eg, those with higher anxiety and depression at pre-treatment) and will examine predictors of treatment efficacy.

### Limitations

This study has certain limitations due to its preliminary nature. The major limitation of this study is its moderate sample size (152 adolescents across 5 classes were randomized). In addition, the adolescents were recruited from only one school, which may limit generalizability of findings. Furthermore, due to financial and personnel constraints, this study only incorporated adolescent self-report measures. Although this methodology facilitates assessment of a large cohort of adolescents in a relatively short time frame [9], it also has limitations (eg, relies on subjective perceptions), and future studies should consider using information from multiple sources (interviews with the students, parents, and teacher reports, etc). In addition, participants were not blinded to allocation when measures were obtained due to constraints established by the Ethical Research Committee, which specified that informed consent forms must state group allocation. Lastly, using the same group leaders to administer the intervention for all classes increases consistency but, at the same time, may limit generalizability of findings [65].

### Conclusions

The current trial will provide insight into the implementation and effectiveness of the UP-A as a universal, school-based anxiety and depression prevention program, adding knowledge to the research base on transdiagnostic prevention programs more generally. This initial cluster RCT was also designed to provide information regarding the feasibility of potential future full-scale trials in order to optimize the intervention and design approach.

## Abbreviations

CASI: Childhood Anxiety Sensitivity Index
CBT: cognitive behavioral therapy
CDN: Depression Questionnaire for Children (Cuestionario de Depresión para Niños)
CONSORT: Consolidated Standards of Reporting Trials
DSM-IV: Diagnostic and Statistical Manual of Mental Disorders, 4th edition
DSM-5: Diagnostic and Statistical Manual of Mental Disorders, 5th edition
EAN: Anxiety Scale for Children (Escala de Ansiedad para Niños)
EASI-A: Emotional Avoidance Strategy Inventory for Adolescents
EBAE-10: School Adjustment Brief Scale (Escala Breve de Ajuste Escolar)
EIDAN: Depression and Anxiety Interference Scale for Children (Escala de Interferencia de la Depresión y Ansiedad para Niños)
ICC: intracluster correlation coefficient
IG: General Indiscipline Scale (Escala de Indisciplina General)
KIDSCREEN-10: Kidscreen-10 Quality of Life Scale
PANASN: Positive and Negative Affect Schedule for Children and Adolescents (Escalas PANAS de Afecto Positivo y Negativo para Niños y Adolescentes)
PID-5-BF: The Personality Inventory for DSM-5-Brief Form
RCT: randomized controlled trial
RCADS-30: Revised Child Anxiety and Depression Scale–30
SDQ: Strengths and Difficulties Questionnaire
SES: Self-Esteem Scale
SMART: specific, measurable, attainable, relevant, and time-bound
SPSS: Statistical Package for the Social Sciences
SWLS-C: Satisfaction with Life Scale for Children
T-CBT: transdiagnostic cognitive behavioral therapy
TPA: Top Problems Assessment
UP: Unified Protocol for Transdiagnostic Treatment of Emotional Disorders
UP-A: Unified Protocol for Transdiagnostic Treatment of Emotional Disorders in Adolescents
UP-C: Unified Protocol for Transdiagnostic Treatment of Emotional Disorders in Children
WL: waitlist
